# Immunogenic Cell Death-Relevant Damage-Associated Molecular Patterns and Sensing Receptors in Triple-Negative Breast Cancer Molecular Subtypes and Implications for Immunotherapy

**DOI:** 10.3389/fonc.2022.870914

**Published:** 2022-04-04

**Authors:** Ming Xu, Jin-hua Lu, Ya-zhen Zhong, Jing Jiang, Yue-zhong Shen, Jing-yang Su, Sheng-you Lin

**Affiliations:** ^1^ Department of Oncology, Hangzhou TCM Hospital Affiliated to Zhejiang Chinese Medical University, Zhejiang, China; ^2^ Department of Traditional Chinese Medicine, The First People’s Hospital of Tongxiang, Zhejiang, China

**Keywords:** triple-negative breast cancer, damage-associated molecular patterns, subtype, immunogenic cell death, immune microenvironment, immunotherapy

## Abstract

**Objectives:**

Triple-negative breast cancer (TNBC) is defined as a highly aggressive type of breast cancer which lacks specific biomarkers and drug targets. Damage-associated molecular pattern (DAMP)-induced immunogenic cell death (ICD) may influence the outcome of immunotherapy for TNBC patients. This study aims to develop a DAMPs gene signature to classify TNBC patients and to further predict their prognosis and immunotherapy outcome.

**Methods:**

We identified the DAMPs-associated subtypes of 330 TNBCs using K-means analysis. Differences in immune status, genomic alterations, and predicted immunotherapy outcome were compared among each subtype.

**Results:**

A total of 330 TNBCs were divided into three subtypes according to DAMPs gene expression: the nuclear DAMPs subtype, featuring the upregulation of nuclear DAMPs; the inflammatory DAMPs subtype, characterized by the gene set enrichment of the adaptive immune system and cytokine signaling in the immune system; and the DAMPs-suppressed subtype, having the lowest level of ICD-associated DAMPs. Among them, the inflammatory subtype patients had the most favorable survival, while the DAMPs-suppressed subtype was associated with the worst prognosis. The DAMPs subtyping system was successfully validated in the TCGA cohort. Furthermore, we systemically revealed the genomic alterations among the three DAMPs subtypes. The inflammatory DAMPs subtype was predicted to have the highest response rate to immunotherapy, suggesting that the constructed DAMPs clustering had potential for immunotherapy efficacy prediction.

**Conclusion:**

We established a novel ICD-associated DAMPs subtyping system in TNBC, and DAMPs expression might be a valuable biomarker for immunotherapy strategies. Our work could be helpful to the development of new immunomodulators and may contribute to the development of precision immunotherapy for TNBC.

## Introduction

Female breast cancer is the most commonly diagnosed cancer and the second leading cause of cancer death worldwide in 2020 (1). Triple-negative breast cancer (TNBC) is a highly aggressive subtype of breast cancer that lacks estrogen receptor (ER), progesterone receptor (PR) and human epidermal growth factor receptor 2 (HER-2) expression (2). Due to its high invasiveness, strong metastatic ability and early recurrence, TNBC causes a larger number of breast cancer-related deaths than other types (3). Nevertheless, recent approvals for immunotherapy have offered potential long-term survival outcomes for metastatic TNBC (mTNBC) patients, regardless of different histological subtypes (4).

Immune checkpoint inhibitors (ICIs), the most successful immunotherapeutic agents for TNBC, can significantly improve patients’ progression-free survival (PFS) and overall survival (OS) if they respond (5). The response rates to ICIs in TNBC are associated with tumor infiltrating lymphocytes (TILs), programmed death ligand-1 (PD-L1) expression and nonsynonymous mutations (6). In addition, preclinical models (7) and clinical trials (8) confirm that the induction of immunogenic cell death (ICD) sensitizes TNBC to ICIs treatments, which suggests that ICD biomarkers may be candidate prognostic factors for immunotherapy.

The major immunogenic characteristic of ICD is defined by the emission of a series of immunostimulatory damage-associated molecular patterns (DAMPs), such as cell surface-exposed calreticulin (CALR), extracellular adenosine triphosphate (ATP) and high mobility group box 1 (HMGB1) (9). Previous studies have demonstrated that TNBC cell-derived HMGB1 elicited by chemo/radiotherapy augmented the antitumor immunity induced by activated CD8^+^ T cells (10, 11), which revealed the importance of DAMPs in treating TNBC with immunotherapy.

Various innate immune receptors are involved in DAMPs-mediated ICD, such as pattern-recognition receptors (PRRs), G protein-coupled receptors (GPCRs), and triggering receptors expressed on myeloid cells (TREMs) (12), and their interaction with DAMPs is necessary for ICD and anticancer immunity. Moreover, the expression patterns of toll-like receptors (TLRs), the most widely studied PRRs, are diverse between TNBC and other types of breast cancer and are associated with different cancer incidence and progression (13). Targeting DAMPs-sensing receptors may provide new insights for the treatment of TNBC, since they have the potential to ignite positive immune responses in the tumor-immune-microenvironment (TIME) (14).

Sensitization to ICIs by ICD-associated DAMPs strongly relies on the immunoreactive TIME, in which TILs and tumor-associated macrophages (TAMs) play an important role in the prognostic prediction of TNBC (15). In this regard, assessing the distinct TIME helps to understand the immune signature of different TNBC subtypes, optimize immunotherapy and improve the outcome of TNBC patients (16). Although a large number of immune-related biomarkers and prognostic models have been developed to describe and quantify the TIME of TNBC (17, 18), few have considered ICD-associated DAMPs and sensing receptors as actionable targets.

**Figure 1:**
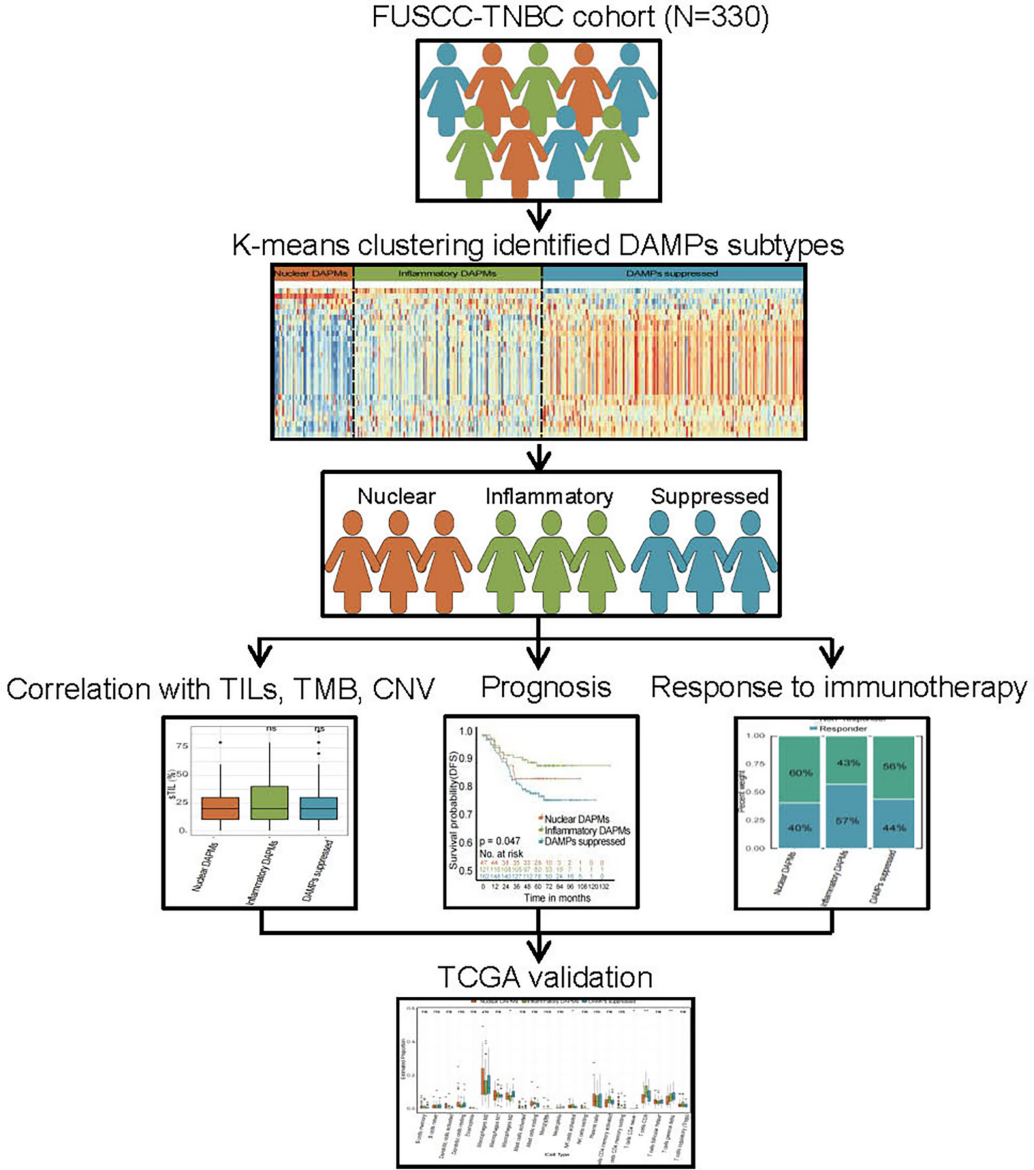
Flow chart of the data analysis process. The DAMPs-associated subtypes were established based on 330 TNBCs from the FUSCC cohort and validated in the TCGA cohort. DAMPs, damage-associated molecular patterns; FUSCC, Fudan University Cancer Center; TCGA, The Cancer Genome Atlas.

In the present study, we comprehensively analyzed the DAMPs gene signature to explore the effect of ICD-associated DAMPs and sensing receptors on the TIME and survival of TNBC patients. Moreover, we constructed a DAMPs-based risk score model to evaluate the prognostic value of DAMPs and correlated sensing receptors in TNBC (the whole process of data analysis is depicted in **Figure 1**). Our work may provide a novel clue for exploring the underlying molecular mechanisms of different responses to immunotherapy in TNBC patients, which sheds a novel light on the immunotherapy strategy of TNBC.

## Materials and Methods

### Data Collection

Our study included cohorts of breast cancer patients from Fudan University Shanghai Cancer Center (FUSCC, a total of 465 TNBC patients, 100% female, average age = 53 ± 11 years; 360 patients with RNA-seq data, 401 patients with CNA data and 279 patients with WES data) (19) and TCGA (a total of 1,096 patients, including 161 TNBC patients, 100% female, average age = 55 ± 12 years) (20–22). To exclude the influence of pathological heterogeneity, we included 330 TNBC samples of invasive ductal carcinoma (IDC) in the FUSCC cohort. The microarray data and sequence data of the FUSCC cohort have been deposited in the NCBI Gene Expression Omnibus (GEO: GSE118527) and Sequence Read Archive (SRA: SRP157974). Expression and clinical data of TCGA cohorts were downloaded from the website (http://www.cbioportal.org/). TNBC sample selection of FUSCC and TCGA was based on the immunohistochemistry (IHC) results, which lack expression of ER, PR, and HER2 in IDC. The demographic data and clinical features are listed in **Table 1**.

**Table 1:** Correlation between clinicopathologic variables and DAMPs associated subtypes.

Variables	Number of patients	DAMPs associated subtypes			P^a^ value
		Nuclear DAMPs	Inflammatory DAMPs	DAMPs suppressed	
Total	330	47	121	162	
Age					0.1357
≤50 years	142	24	57	61	
>50years	188	23	64	101	
Menopausal status					0.0725
Premenopause	120	23	47	50	
Postmenopause	206	24	73	109	
Tumor size stage					0.6244
T1	121	14	43	64	
T2	204	32	77	95	
T3	4	1	1	2	
Lymph node status					0.4762
Negative	188	27	74	87	
Positive	140	19	47	74	
Grade					0.9447
2	61	9	23	29	
2 to 3	28	3	9	16	
3	228	34	84	110	
FUSCC TNBC subtype					0.0020
BLIS	126	25	45	56	
IM	81	8	43	30	
LAR	73	10	19	44	
MES	50	4	14	32	

FUSCC, Fudan University Shanghai Cancer Center; TNBC, triple-negative breast cancer; BLIS, basal-like immune suppressed; LAR, luminal-androgen receptor; IM, immunomodulatory; MES, mesenchymal-like.

^a^Based on Fisher’s exact method.

### Identification of DAMPs Subgroups by K-means Analysis

First, 32 DAMPs-related genes were collected according to previous research, and their information is shown in **Table 2** (9, 12, 23, 34). 28 DAMPs-related genes were found to be expressed in the FUSCC cohort. Consensus clustering was analyzed by the R package “ConsensusClusterPlus” based on the DAMPs-related gene expression matrix. Consist with previous FUSCC TNBC research, k-means clustering (the “kmeans” function in R) was utilized to determine stable DAMPs-associated TNBC subtypes (19, 24).

**Table 2:** DAMPs-related genes (9, 12, 23, 34).

Genes	Molecular type	Expression pattern of protein	Function
CALR	DAMPs	Endoplasmic reticulum	Tumor cell uptake by DCs and chemotherapy-induced antitumoral immune response
HMGB1	DAMPs	Translocates from the nucleus to the cytoplasm upon autophagy stimulation	Promotes the maturation and cross-presentation activity of APCs
HMGN1	DAMPs	Cell nucleus and cytoplasm	Induces dendritic cell maturation, recruitment of APCs and antigen-specific immune responses
IL1A	DAMPs	Mesothelial cells	Cell activation, cytokine release
IL33	DAMPs	Intracellular	Can bind ST2 on mast cells and TH2 cells and trigger secretion of pro-inflammatory and TH2 cyokines. The immunostimulatory activity of IL-33 might be inactivated during apoptosis
ROCK1	DAMPs	Cytoplasm	Release the find-me signals ATP and UTP
PANX1	DAMPs	Cell membrane	Release the find-me signals ATP and UTP
BCL2	DAMPs	Nucleus membrane	Reduces reperfusion injury of skeletal or cardiac muscle when injected extracellularly
PPIA	DAMPs	Intracellular	Initiate and perpetuate the inflammatory response
HSPA4	DAMPs	Cytoplasm	Protein folding, protein refolding, protein transport, and protein targeting
HSP90AA1	DAMPs	Cytoplasm	Provides chaperoning activity for client proteins
TLR2	Receptor (TLRs)	Ubiquitous, high in DCs, monocytes, macrophages and neutrophils	Promotes the production of pro-inflammatory cytokines and chemokines
TLR3	Receptor (TLRs)	Ubiquitous, high in DCs, monocytes, macrophages and NK cells	Promotes the production of pro-inflammatory cytokines, chemokines and IFN-I
TLR4	Receptor (TLRs)	Ubiquitous, high in DCs, monocytes, macrophages, neutrophils and endothelial cells	Promotes the production of pro-inflammatory cytokines, chemokines and IFN-I
TLR7	Receptor (TLRs)	Ubiquitous, high in pDCs, monocytes, macrophages and B cells	Promotes the production of IFNα and other cytokines and chemokines
TLR9	Receptor (TLRs)	Ubiquitous, high in pDCs, monocytes, macrophages and B cells	Promotes the production of IFNα and other cytokines and chemokines
CLEC4E	Receptor	Monocytes, macrophages, DCs, neutrophils and B cells	Promotes the release of pro-inflammatory cytokines
CLEC7A	Receptor	Monocytes, macrophages, DCs, neutrophils, mast cells, T and B cells	Initiating of intracellular signalling that produce pro-inflammatory cytokines
NLRP3	Receptor (NLRs)	DCs, neutrophils, monocytes and macrophages	Promotes IL-1β and IL-18 secretion and initiates pyroptosis
DDX58	Receptor	Ubiquitous, highly expressed in epithelial cells and myeloid cells	Trigger a transduction cascade which inducting the expression of antiviral cytokines
IFIH1	Receptor	Cytoplasm, nucleus	Promotes the production of IFN-I and other cytokines and chemokines
CGAS	Receptor (CDSs)	Ubiquitous, highly expressed in epithelial cells, DCs, monocytes, macrophages and T cells	Promotes the production of IFN-I and other cytokines and chemokines
AIM2	Receptor (CDSs)	Ubiquitous, highly expressed in epithelial cells, DCs, monocytes, macrophages, B cells and NK cells	Promotes IL-1β and IL-18 secretion and initiates pyroptosis
AGER	Receptor	Ubiquitous	Promotes the expression of pro-inflammatory genes, as well as cell migration, proliferation and apoptosis
TREM1	Receptor (TREMs)	Myeloid cells, epithelial cells, endothelial cells and fibroblasts	Promotes pro-inflammatory cytokine and chemokine secretion
FPR1	Receptor (GPCRs)	Ubiquitous, high in neutrophils, monocytes and macrophages	Promotes chemotaxis of neutrophils and monocytes/macrophages
FPR2	Receptor (GPCRs)	Ubiquitous, high in neutrophils, monocytes and macrophages	Promotes chemotaxis of neutrophils and monocytes/macrophages
P2Y2R	Receptor (GPCRs)	Ubiquitous, high in epithelial cells, neutrophils, DCs, monocytes and macrophages	Promotes migration and activation of various immune cells
P2Y6R	Receptor (GPCRs)	Ubiquitous, high in stromal cells, neutrophils, monocytes, macrophages and T cells	Promotes proliferation and cytokine and chemokine production in stromal cells
P2Y12R	Receptor (GPCRs)	Mainly in platelets, also in DCs, monocytes, macrophages and T cells	Promotes platelet activation and Th17 differentiation
CASR	Receptor (GPCRs)	Ubiquitously expressed	Promotes monocyte/macrophage recruitment and NLRP3 activation
P2RX7	Receptor (Ion channels)	Ubiquitous	Promotes cytokine and chemokine production, NLRP3 inflammasome activation and T cell activation

DAMPs, damage-associated molecular patterns; CALR, calreticulin; DCs, dendritic cells; HMGB1, high mobility group box 1; APCs, antigen-presenting cells; HMGN1, high-mobility group nucleosome binding protein 1; IL, interleukin; ROCK1, Rho-associated coiled-coil containing protein kinase 1; PANX1, pannexin 1; ATP, adenosine triphosphate; UTP, Uridine 5’-triphosphate; BCL2,B-cell lymphoma-2; PPIA, cyclophilin-A; HSP, heat shock protein; TLRs, toll-like receptors; NK, natural killer cell; IFN, interferon; pDCs, plasmacytoid dendritic cells; CLEC, C-type lectin receptor; NLRP3, NACHT, leucine-rich repeat and pyrin domains-containing protein 3; NLRs, NOD-like receptors; DDX58, DExD/H-box helicase 58; IFIH1, interferon induced with helicase C domain 1; CGAS, cyclic GMP-AMP synthase; CDSs, cytosolic DNA sensors; AIM2, absent in melanoma 2; AGER, advanced glycosylation end product-specific receptor; TREM, triggering receptors expressed on myeloid cells; FPR, N-formyl peptide receptor; GPCRs, G protein-coupled receptors; P2Y2R, P2Y2 receptor; P2Y6R, P2Y6 receptor; P2Y12R, P2Y12 receptor; CASR, calcium-sensing receptor; P2RX7, Purinergic Receptor P2X 7.

### PAM50 Subtype and Lehmann TNBC Subtype

The PAM50 subtype of FUSCC TNBC was analyzed by the PAM50 predictor (the “genefu” package in R). To identify Lehmann TNBC molecular subtypes, only TNBC samples that were determined by mixed Gaussian distribution were subtyped as individual datasets using the TNBC type online subtyping tool (http://cbc.mc.vanderbilt.edu/tnbc/) (25).

### Functional Analyses

Differentially expressed genes (DEGs) among the three subtypes were identified by the threshold value of |log2FC|≥1 and false discovery rate (FDR) ≤0.05. Gene Ontology (GO) analysis and Kyoto Encyclopedia of Genes and Genomes (KEGG) analysis were performed and visualized in Metascape (26). Furthermore, gene set enrichment analysis (GSEA) was utilized among DAMPs-associated TNBC subtypes to compare the differences (27).

### Genomic Analysis

Copy number alterations (CNA) were analyzed using GISTIC2.0 from the FUSCC TNBC cohort (28). We compared the differences in amplification or deletion events at the arm level among DAMPs-associated TNBC subtypes. The waterfall plot of CNV data was visualized by the “ComplexHeatmap” package in R. The tumor mutation burden (TMB) was calculated by the number of mutations per patient (29).

### Immune Analyses

CIBERSORT immune infiltrating analysis was performed to calculate the abundance of 22 immune infiltrating cells (30). We calculated the stromal score and immune score through the Estimation of Stromal and Immune cells in Malignant Tumor tissues using Expression data (ESTIMATE) algorithm (31).

### Calculation of the Immunophenscore

The immunophenoscore (IPS) was calculated by the algorithm as previously reported (32). Briefly, IPS was calculated on a 0-10 scale based on the expression of the representative genes or gene sets of the immunophenogram. Samplewise z scores are positively weighted according to stimulatory factors (cell types) and negatively weighted according to inhibitory factors (cell types) and averaged. Z scores ≥3 were designated IPS10, and z scores ≤0 were designated IPS0.

### Statistical Analyses

Statistical analyses were conducted by R (version 3.5.2) and GraphPad Prism (version 8.0). Survival analysis was performed through the Kaplan–Meier algorithm by the R package “survival”. Two-sided Student’s t test or two-sided Wilcoxon-rank sum test were used for comparison of two groups. For comparison of more than three groups, one-way analysis of variance (ANOVA) or Kruskal-Wallis test were performed. A *P* < 0.05 was considered statistically significant.

## Results

### Identification of Three DAMPs-Associated Subtypes

**Figure 2:**
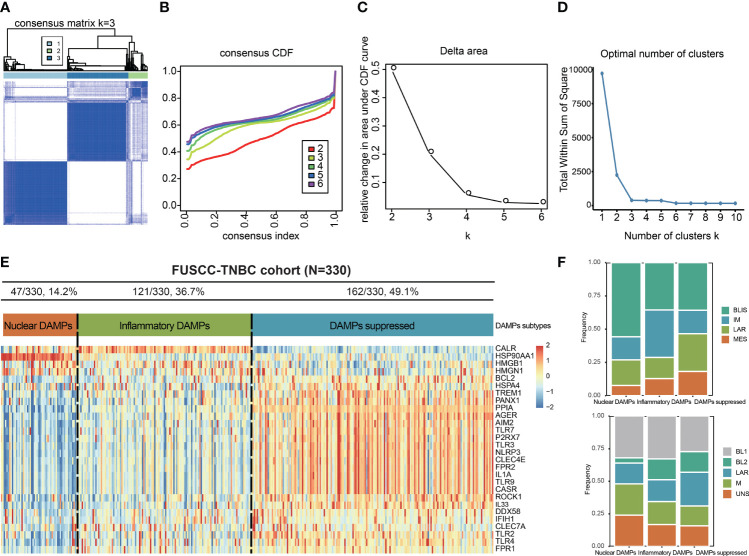
Identification of DAMPs-associated subtypes by K-means analysis. **(A-C)** K = 3 was identified as the optimal value for consensus clustering. **(D)** K = 3 was identified as the optimal value for cluster sums of squares. **(E)** DAMPs-associated subtyping of TNBC samples (n = 330) in the FUSCC cohort. Heatmap shows normalized enrichment scores of the three DAMPs-associated subtypes. **(F)** Bar plots showing the distribution of FUSCC TNBC subtypes and Lehmann TNBC subtypes among the DAMPs-associated subtypes.

To exclude the influence of pathological heterogeneity, we included TNBC samples of IDC (n=330) from the FUSCC cohort. We classified them into three DAMPs-associated subtypes based on the expression of DAMPs-related genes by a consensus clustering approach, identifying the optimal clustering stability at K=3 (**Figures 2A-D**). Of the 330 TNBC patients included in the study, 47 patients were clustered in the nuclear DAMPs subtype, 121 in the inflammatory DAMPs subtype and 162 in the DAMPs-suppressed subtype. The heatmap shows the normalized enrichment scores of DAMPs-related genes in the three subtypes (**Figure 2E**), with significant differences in expression between the three distinct clusters. Nuclear-associated DAMPs, such as HMGB1 and high-mobility group nucleosome binding protein 1 (HMGN1), were highly expressed in the nuclear DAMPs subtype. The inflammatory DAMPs subtype was characterized by high expression of CALR. In the DAMPs-suppressed subtype, DAMPs genes were expressed at low levels, but DAMPs receptors were highly expressed (**Figure 2E**). Stacked bar plots show the distribution of FUSCC TNBC subtypes and Lehmann TNBC subtypes among the DAMPs-associated subtypes (**Figure 2F**), demonstrating that the immunomodulatory (IM) subtypes of four FUSCC TNBC subtypes (luminal androgen receptor/IM/basal-like immune-suppressed/mesenchymal-like) account for the highest proportion of the inflammatory DAMPs subtype. These results suggest that DAMPs-related genes classify TNBC patients into three distinguishable subtypes with significantly different gene expression patterns, as well as different predominant clinical subtypes.

### Differentially Expressed Gene Analysis and Functional Analyses

**Figure 3:**
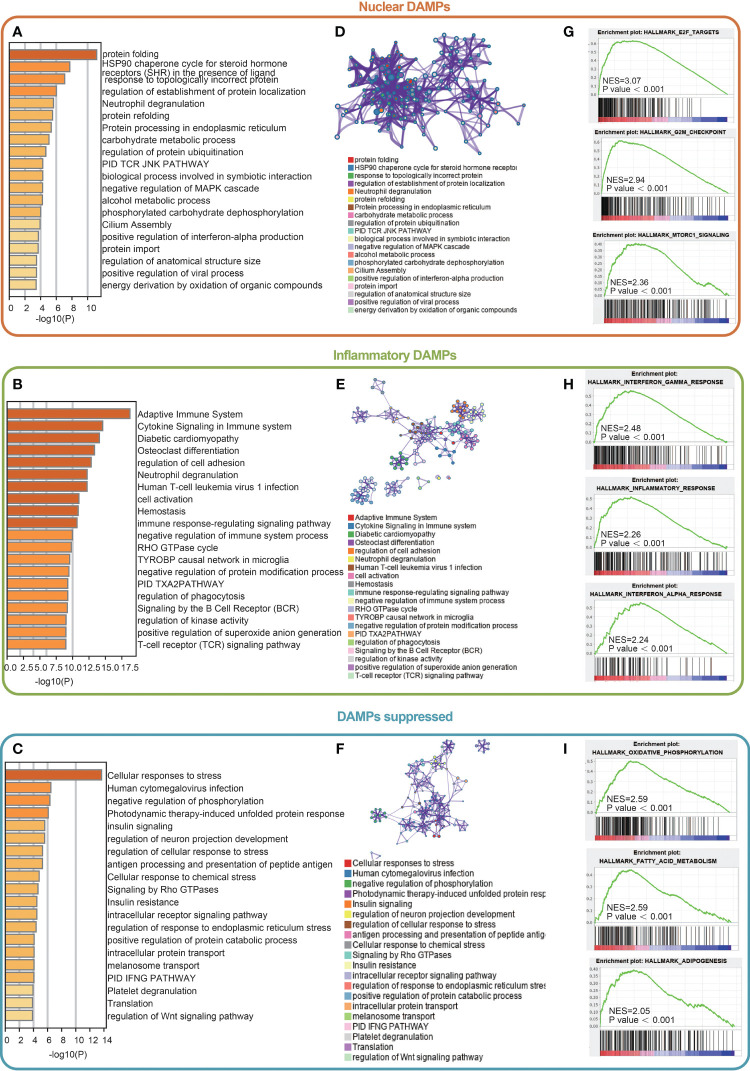
Differentially expressed gene (DEG) analysis and functional analyses. **(A-C)** Bar diagram showing the signaling pathways enriched by Kyoto Encyclopedia of Genes and Genomes (KEGG) analysis. **(D-F)** Circle plot and network visualizing the biological processes enriched by gene ontology (GO) analysis. **(G-I)** Gene set enrichment analysis (GSEA) results showing the activated signaling pathways in the DAMPs-associated subtypes.

We next identified DEGs among the three subtypes and performed functional analysis to explore their potential signaling mechanisms. A total of 1,406 DEGs were detected, of which 166 were in the nuclear DAMPs subtype, 893 were in the inflammatory DAMPs subtype and 347 were in the DAMPs-suppressed subtype. For the nuclear DAMPs subtype, KEGG enrichment analysis revealed that the DEGs were mainly enriched in heat shock protein (HSP)-related signaling pathways, including protein folding, the HSP90 chaperone cycle for steroid hormone receptors (SHRs), the presence of ligands and neutrophil degranulation (**Figure 3A**). Likewise, GO enrichment analysis also showed that the DEGs were enriched in HSP-related signaling pathways (**Figure 3D**), indicating that HSPs play an important role in the presentation of tumor antigens. Consistently, GSEA showed that the nuclear DAMPs subtype proliferates vigorously. (**Figure 3G**). For the inflammatory DAMPs subtype, KEGG enrichment analysis revealed that the DEGs were significantly enriched in immune-related signaling pathways, including the adaptive immune system, cytokine signaling in the immune system, neutrophil degranulation, the immune response-regulating signaling pathway, signaling by the B cell receptor (BCR), and the T cell receptor (TCR) signaling pathway (**Figure 3B**). Similarly, GO enrichment analysis also identified that the DEGs were enriched in immune-related signaling pathways (**Figure 3E**). GSEA revealed that the interferon gamma (INF-γ) response, inflammatory response, and interferon alpha (INF-ɑ) response differentiation were highly expressed in the inflammatory DAMPs subtype (**Figure 3H**). For the DAMPs-suppressed subtype, KEGG enrichment analysis revealed that the DEGs were notably upregulated in cell stress-related pathways, including cellular responses to stress and negative regulation of phosphorylation (**Figure 3C**). GO analysis also presented a similar result (**Figure 3F**). GSEA showed a significant upregulation in oxidative phosphorylation, fatty acid metabolism and adipogenesis (**Figure 3I**).

### Comparison of Genomic Alterations of DAMPs-Associated Subtypes in the FUSCC-TNBC Cohort

**Figure 4:**
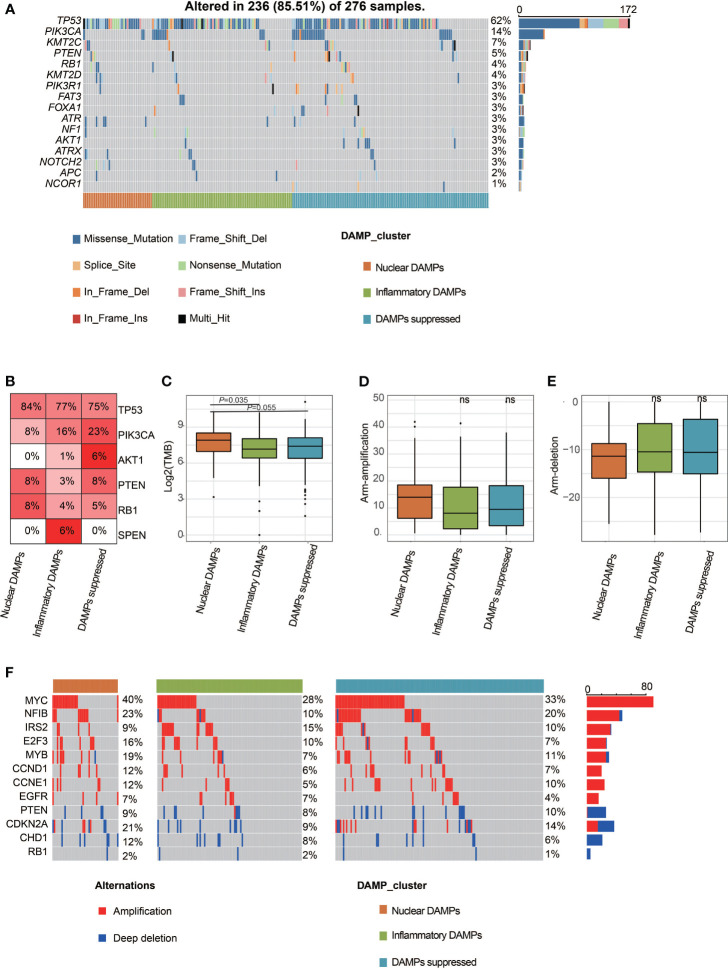
Comparison of genomic alterations of DAMPs-associated subtypes in the FUSCC-TNBC cohort. **(A)** Differential somatic mutation analysis among the three subgroups. **(B)** Somatic mutation percentage of mostly mutated genes. **(C)** Tumor mutant burden difference among the three subtypes in the FUSCC cohort. **(D)** Arm-level copy number amplification in DAMPs-associated subtypes. **(E)** Arm-level copy number deletion in DAMPs-associated subtypes. **(F)** Distinct CNA profile among the three subgroups. ns, not significant.

We used oncoplot to identify genomic alterations among the three DAMPs-associated subtypes in the FUSCC-TNBC cohort, and the results showed that PI3K/AKT pathway mutations were least frequently observed in nuclear DAMPs subtypes and most commonly observed in DAMPs-suppressed subtypes (*P*=0.0037; **Figures 4A, B**), which may be related to the fact that DAMPs-suppressed subtypes contain more luminal/androgen receptor (LAR) subtypes. Interestingly, split ends (SPEN) mutations were only observed in the inflammatory DAMPs subtype. The TMB of the nuclear DAMPs subtype was higher than that of the remaining two subtypes (**Figure 4C**). Although the frequency of CNV in the nuclear DAMPs subtype was also higher than that in the other two subtypes, it failed to achieve statistical significance (**Figures 4D–F**).

### Patients in the Three Molecular Subtypes Exhibited Different Immune Statuses

**Figure 5:**
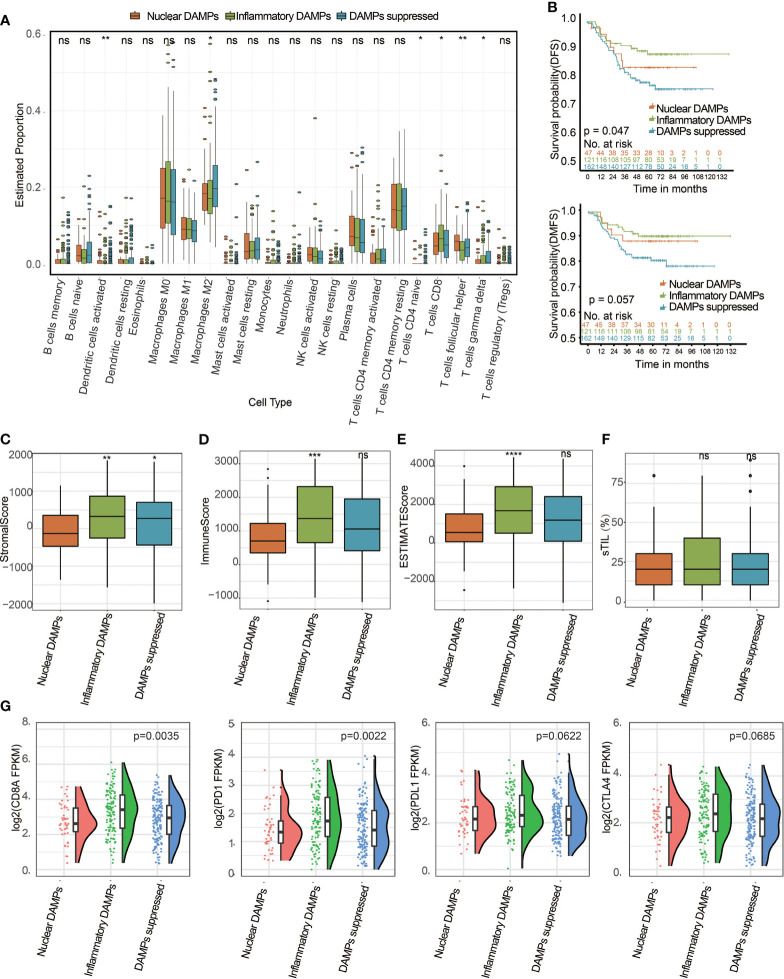
Identification of DAMPs-associated subtypes among the FUSCC-TNBC cohort and comparison of their differences in tumor-infiltrating lymphocyte immune biomarker expression levels. **(A)** The differential estimated proportion of 22 CIBERSORT immune cell types in DAMPs-associated subtypes. The central line represents the median value. The bottom and top of the boxes are the 25th and 75th percentiles (interquartile range). The whiskers encompass 1.5 times the interquartile range. **(B)** Kaplan-Meier curves of disease-free survival (DFS) and distant metastasis-free survival (DMFS) among the three subtypes in the FUSCC cohort. **(C)** Stromal score in DAMPs-associated subtypes. **(D)** Immune score in DAMPs-associated subtypes. **(E)** ESTIMATE score difference among the three subtypes in the FUSCC cohort. **(F)** The sTIL difference among DAMPs-associated subtypes. **(G)** Expression differences in CD8A, PD-1, PD-L1 and CTLA4 among the three subtypes. *P < 0.05; **P < 0.01; ***P < 0.001; ****P < 0.0001; ns, not significant.

Immune analyses were conducted to explore the immune differences between the three subtypes. The CIBERSORT algorithm revealed that CD8^+^ T cells in the inflammatory DAMPs subtype increased significantly, while M2-like macrophages increased significantly in the DAMPs-suppressed subtype (**Figure 5A**). Kaplan–Meier survival analysis revealed that patients in the inflammatory DAMPs subtype had the best disease-free survival (DFS) and distant metastasis-free survival (DMFS), while patients in the DAMPs-suppressed subtype tended to have the worst outcome. (DFS: *P*=0.047, DMFS: *P*=0.057; **Figure 5B**). Furthermore, the ESTIMATE algorithm revealed that patients in the inflammatory DAMPs subtype had significantly higher stromal scores, immune scores and ESTIMATE scores than the others, with no significant difference found in sTILs (**Figures 5C–F**). Interestingly, however, the prognosis of the nuclear DAMPs subtype is better than that of the DAMPs-suppressed subtype, despite having the lowest immune score. In addition, the expression of CD8A (*P*=0.0035) and PD-1 (*P*=0.0022) in the inflammatory DAMPs subtype was significantly higher than that in the other two subtypes, while no statistical significance was detected regarding the expression of PD-L1 (*P*= 0.0622) and CTLA4 (*P*=0.0685; **Figure 5G**), which suggested that PD-L1 and CTLA4 may not be the best markers of PD-1 efficacy in TNBC.

### Successful Validation of the Immunophenotypes in the TCGA Cohort

**Figure 6:**
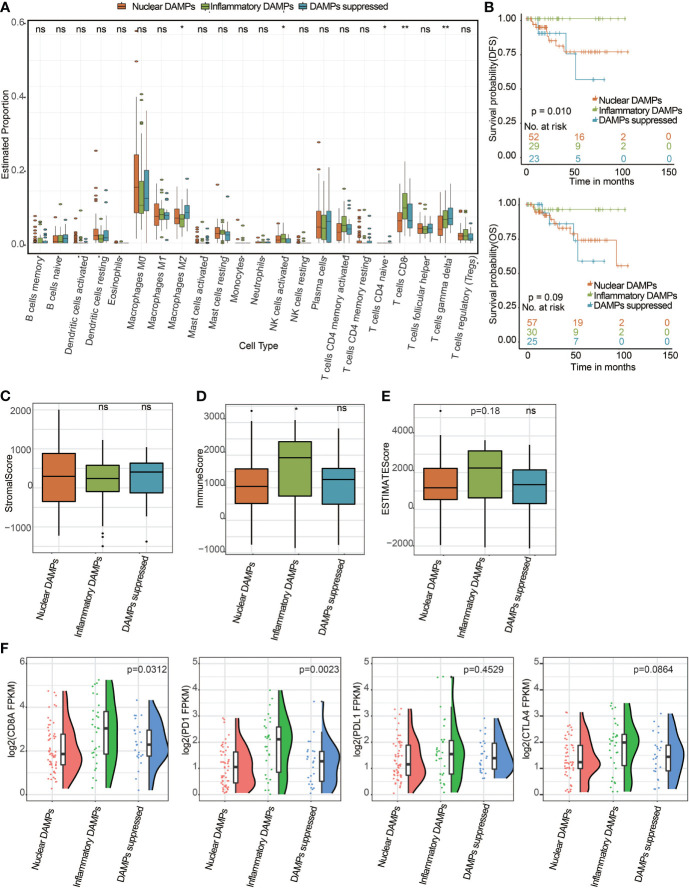
Successful validation of DAMPs-associated subtypes in the TCGA cohort. **(A)** The differential estimated proportion of 22 CIBERSORT immune cell types in DAMPs-associated subtypes. The central line represents the median value. The bottom and top of the boxes are the 25th and 75th percentiles (interquartile range). The whiskers encompass 1.5 times the interquartile range. **(B)** Kaplan-Meier curves of overall survival (OS) and disease-free survival (DFS) among the three subtypes in the TCGA-TNBC cohort. Arm-level copy number amplification in DAMPs-associated subtypes. **(C)** Stromal score in DAMPs-associated subtypes. **(D)** Immune score in DAMPs-associated subtypes. **(E)** ESTIMATE score difference among the three subtypes in the TCGA cohort. **(F)** Expression differences in CD8A, PD-1, PD-L1 and CTLA4 among the three subtypes. *P < 0.05; **P < 0.01; ns, not significant.

To further validate whether the immune differences we found among the different subtypes of the FUSCC cohort could be generalized, we selected TNBC patients from the TCGA public database and divided them into 3 DAMPs-associated subtypes. According to the CIBERSORT algorithm as above, we found that CD8^+^ T cells in the inflammatory DAMPs subtype increased significantly and M2-like macrophages in the DAMPs-suppressed subtype increased significantly, which is similar to the results of the FUSCC cohort (**Figure 6A**). Survival analysis also revealed that patients in the inflammatory DAMPs subtype enjoyed the best OS and DFS, while patients in the DAMPs-suppressed subtype had the worst (DFS: *P*=0.010, OS: *P*=0.09; **Figure 6B**). Additionally, patients in the inflammatory DAMPs subtype had higher immune scores than others (**Figure 6D**), while no significant difference was found in stromal scores and ESTIMATE scores (**Figures 6C, E**). In addition, the expression of CD8A (*P*=0.0312) and PD-1 (*P*=0.0023; **Figure 6F**) in the inflammatory DAMPs subtype from the TCGA cohort was notably higher than that in the other two subtypes. The expression of PD-L1 and CTLA4 was similar to the trend in the FUSCC cohort, which again indicated that PD-L1 and CTLA4 may not be the best markers of PD-1 efficacy in TNBC.

### DAMPs-Associated Subtypes Can Predict Immunotherapy Outcome

**Figure 7:**
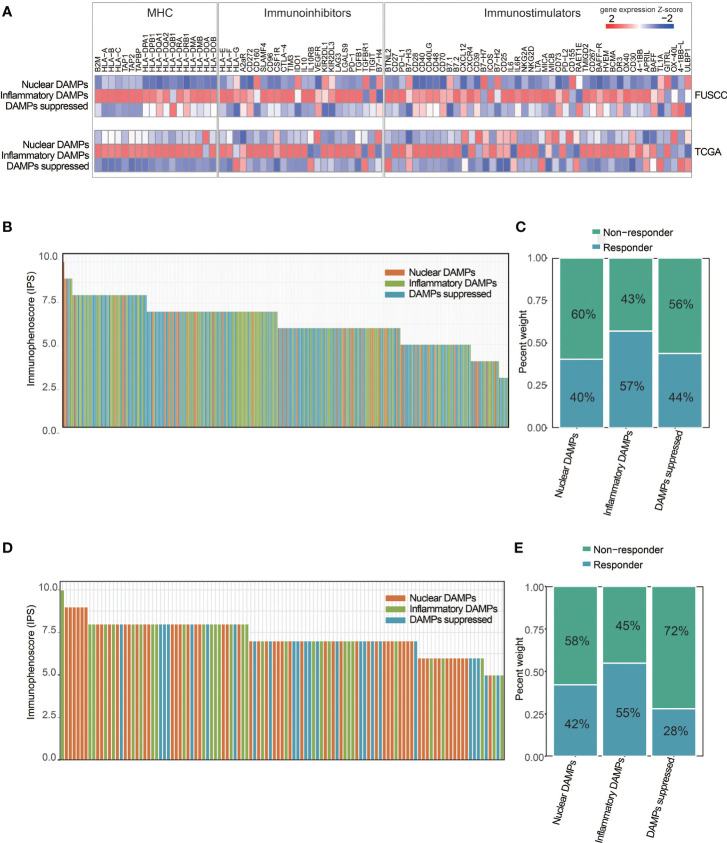
DAMPs-associated clusters can predict immunotherapy outcome. **(A)** Expression of MHC and immunomodulatory molecules for DAMPs-associated subtypes. Expression values are represented by z scores calculated across all tumors and color coded according to the legend. **(B)** Waterfall plot illustrating the Immunophenoscores (IPS) according to the DAMPs-associated subtypes in the FUSCC cohort. **(C)** Proportion of patients: Responder and Nonresponder: 40%/60% in the nuclear DAMPs subtype, 57%/43% in the inflammatory DAMPs subtype and 44%/56% in the DAMPs-suppressed subtype. **(D)** Waterfall plot illustrating the IPS according to DAMPs-associated subtypes in the TCGA-TNBC cohort. **(E)** Proportion of patients: Responder and Non−responder: 42%/58% in the nuclear DAMPs subtype, 55%/45% in the inflammatory DAMPs subtype and 28%/72% in the DAMPs-suppressed subtype.

We next explored the predictive ability of immunotherapy outcome across different DAMPs-associated subtypes. First, the expression of major histocompatibility complex (MHC) and immunomodulatory molecules for DAMPs-associated subtypes is shown *via* the heatmap (**Figure 7A**), which revealed that MHC molecules and immunostimulatory and immunoinhibitory molecules are differentially expressed in different subtypes, with the highest expression in the inflammatory DAMPs subtype, both in FUSCC and in TCGA. In addition, the IPS algorithm was performed to evaluate the IPS and the response rate of the three subtypes from FUSCC. The results revealed that the inflammatory DAMPs subtype had the highest response rate (57%; **Figures 7B, C**). Similarly, we used TNBC patients from TCGA as the validation cohort to test the stability of the above findings, and the results showed that the inflammatory DAMPs subtype also had the highest response rate (55%; **Figures 7D, E**). These results suggested that the constructed DAMPs-associated clusters had potential for the prediction of PD-1 treatment efficacy in TNBC.

## Discussion

TNBC is a highly invasive subtype of breast cancer and is an extremely difficult challenge for treatment due to its relatively low response to conventional therapeutics. Despite the booming development of immunotherapy and promising results of ICIs to treat TNBC, it still leads to high mortality in women worldwide (33). Therefore, the current major challenges are how to improve the response of TNBC patients to ICIs treatment and recognize ICIs nonresponders and responders before treatment. Since the emission of ICD-associated DAMPs and their interactions with innate immune receptors play an important role in the activation of anticancer immunity (34), DAMPs and sensing receptors are potential predictors of the response to ICIs treatments in TNBC. In the present study, we identified three molecular subtypes (nuclear/inflammatory/suppressed) based on the gene expression of DAMPs and sensing receptors, which exhibited significantly different DAMPs landscapes. Functional analyses of DEGs revealed that upregulation of immune (INF-γ) and HSP (HSP90) relative genes was implicated with a better prognosis. Further immune analyses indicated that patients with a better prognosis were in a relatively high immune status (CD8A^high^, PD-1^high^) and possessed a higher immune score and ESTIMATE score. Moreover, we established a novel prognostic risk model based on DAMPs and sensing receptors, which predicted the immunotherapy outcome of TNBC patients in FUSCC and TCGA. Our results may meet the need of precision immunotherapy designing for TNBC.

It is becoming increasingly clear that DAMPs show robust adjuvanticity during ICD procedures (35), thus enhancing the antitumor immune effect of ICIs therapy (36). Therefore, we propose here to identify ICD-activating or ICD-suppressive DAMPs subtypes or hallmarks to explore the potential role of a combined DAMPs and immune status classifier for TNBC. In our study, 330 TNBC patients were divided into three DAMPs subtypes. Among them, nuclear-associated HMGB1 and HMGN1 were highly expressed in the nuclear DAMPs subtype, while CALR was highly expressed in the inflammatory DAMPs subtype. HMGB1 and HMGN1 are important nuclear DAMPs, and their translocation to the cytoplasm can activate proinflammatory signaling by binding to PRRs, resulting in TIL influx in breast cancer (37). As an endoplasmic reticulum (ER) marker, when exposed on the tumor cell surface during ICD, CALR promotes the uptake of tumor-specific antigen (TSA), and its mutation compromises immunosurveillance (38). In animal models, the decreased expression of CALR and increased expression of PD-L1 on TNBC cells are associated with fewer CD8^+^ cytotoxic T cells and more regulatory T cells (39).

Recently, another study of FUSCC TNBC subtypes showed that the basal-like immune-suppressed (BLIS) subtype exhibits high genomic instability, and PI3K signaling pathway mutations are observed in the LAR subtype, while immune cell signaling and TILs are elevated in the IM subtype (19). In our classification, the IM subtype is the major inflammatory DAMPs subtype, while the BLIS subtype has the highest frequency of nuclear DAMPs, and the DAMPs-suppressed subtype contains more LAR subtypes. Interestingly, mutations of SPEN, an estrogen receptor (ERα) corepressor, were found in the inflammatory DAMPs subtype. A previous study showed that high SPEN expression is associated with early metastasis in patients with ERα-negative breast cancer (40), while its relationship with DAMPs is still unclear. In addition, a high level of TMB is a characteristic of nuclear DAMPs, which are potential biomarkers of genomic instability and ICIs response (41). Similarly, we found that PI3K/AKT pathway mutations were mostly observed in the DAMPs-suppressed subtype, and it has been reported that dysfunction of the PI3K/AKT pathway is associated with suppressed DAMPs release (42).

The tumor microenvironment (TME), TILs and TAMs play a crucial role in tumor progression, ICIs response and patient outcomes in TNBC (43). In our study, the CIBERSORT and ESTIMATE algorithms revealed that TNBC patients in the three molecular subtypes exhibited different immune statuses. The CIBERSORT algorithm indicated that the inflammatory DAMPs subtype had the largest proportion of CD8^+^ T cells compared with the other two subtypes and was associated with the best predicted outcome. However, we found that M2-like macrophages in the DAMPs-suppressed subtype increased significantly. M2-like macrophages have been recently reported to lead to resistance to ICIs therapy (44) and are associated with unfavorable prognosis in TNBC patients (45). The ESTIMATE algorithm showed that patients with this subtype had significantly higher stromal scores, immune scores and ESTIMATE scores than the remaining two subtypes. Moreover, the significantly higher expression of CD8A and PD-1 in the inflammatory DAMPs subtype may be related to its better DFS and DMFS. These observations are in line with previous reports that TNBC patients with elevated CD8A (46) and PD-1 (47) expression can potentially benefit from ICIs. Additionally, these results were successfully validated with the TCGA public database.

Furthermore, functional analyses were conducted to explore the underlying biological mechanisms. Based on the identified DEGs, GO analysis and KEGG analysis synergistically suggested that DEGs in the inflammatory DAMPs subtype were significantly enriched in the adaptive immune system and cytokine signaling in the immune system. GSEA further elucidated that INF-γ response expression in the inflammatory DAMPs subtype was higher than that in the other two subtypes. INF-γ is exclusively involved in both innate and adaptive immune responses against tumor cells (48). For the nuclear DAMPs subtype, KEGG and GO enrichment analyses both revealed that the DEGs were mainly enriched in HSP-related signaling pathways, such as the HSP90 chaperone cycle. Several studies have demonstrated that the cell surface exposure of HSP90 followed by chemotherapy is correlated with dendritic cell (DC) maturation, resulting in the enhancement of the anticancer immune response (49, 50). For the DAMPs-suppressed subtype, KEGG and GO enrichment analyses indicated that the DEGs were largely upregulated in cell stress-related pathways, and GSEA further showed a significant upregulation in adipogenesis. Since TNBC patients with high adipogenesis have low CD8^+^ T cell and high M2 macrophage infiltration (51), this finding explains why the DAMPs-suppressed subtype has a worse outcome.

As mentioned above, although high TIL, PD-1 or PD-L1 expression indicates that patients are more likely to respond to immunotherapy, these biomarkers fit only a minority of the responders. Therefore, the development of prognostic models that enable the stratification of patients into responders and nonresponders to immunotherapy is urgently needed. In our work, the expression of MHC molecules (class I, class II, and nonclassical), immunostimulators and immunoinhibitors were analyzed, and an immunophenoscores algorithm was performed to predict the immunotherapy outcome. The results revealed that the inflammatory DAMPs subtype had the highest expression of MHC and immunomodulators, along with the highest response rate, which was further validated in the TCGA cohort. MHC molecules, especially human leukocyte antigen class I (HLA-I) alleles, play a critical role in anticancer immunity by recognizing tumor-specific antigens, and their heterozygosity influences patient survival after ICIs treatments (52). Several immunomodulators are involved in immune destruction and tumor escape. Evidence shows that higher expression of immunostimulators is associated with a higher vulnerability to ICIs in breast cancer (53). These results suggested that there is a possible relationship between DAMPs-associated hallmarks and MHC molecules and immunomodulators, which shows good potential for predicting ICIs treatment efficacy.

Consistent with our results, as mediators of tumor immunogenicity, ICD-associated DAMPs and the sensing receptor landscape were correlated with the TIME. This landscape deserves serious attention in determining the immunotherapy approach for TNBC patients. It is also worth noting that ICD-associated DAMPs and sensing receptors have been mentioned more frequently in recent studies regarding the TIME, which contributes to a better understanding of TNBC subtype classification (54). However, limitations and drawbacks exist in our researches. Since ATP is not a gene like CALR or HMGB1, we cannot analyze ATP directly through the transcriptome. We also included ATP receptor molecules in the DAMPs-related gene list. However, these molecules had no significant effect on clustering of DAMPs-associated subtypes. Further studies are warranted to validate the clinical significance of DAMPs-associated molecules and the mechanisms involved in TNBC immunomodulation.

## Conclusion

In conclusion, in the present study, three molecular subtypes were identified based on K-means analysis in TNBC. Immune analysis and functional analyses revealed that the expression of ICD-associated DAMPs and sensing receptors may determine tumor immunogenicity, thereby resulting in good prognosis. Our work could shed a novel light on the development of new immunomodulators that target ICD-associated DAMPs and sensing receptors and may facilitate the development of precision immunotherapy for TNBC.

## Data Availability Statement

The original contributions presented in the study are included in the article/supplementary material. Further inquiries can be directed to the corresponding author.

## Ethics Statement

Ethical review and approval were not required for the study on human participants in accordance with the local legislation and institutional requirements. The patients/participants provided their written informed consent to participate in this study.

## Author Contributions

MX was responsible for data analysis and writing of the draft of the manuscript. J-HL, Y-ZZ and JJ researched the data. Y-ZS and J-YS evaluated the images and contributed to the discussion. S-YL reviewed/edited the manuscript. All authors contributed to the article and approved the submitted version.

## Funding

The study was supported by the construction fund of medical key disciplines of Hangzhou (OO20200385), Zhejiang Lin Shengyou famous traditional Chinese medicine expert inheritance studio project (GZS202002), and the science and technology planning projects of Zhejiang Province (2018C03025).

## Conflict of Interest

The authors declare that the research was conducted in the absence of any commercial or financial relationships that could be construed as a potential conflict of interest.

## Publisher’s Note

All claims expressed in this article are solely those of the authors and do not necessarily represent those of their affiliated organizations, or those of the publisher, the editors and the reviewers. Any product that may be evaluated in this article, or claim that may be made by its manufacturer, is not guaranteed or endorsed by the publisher.
